# HW_TEST, a program for comprehensive HARDY-WEINBERG equilibrium testing

**DOI:** 10.1590/1678-4685-GMB-2019-0380

**Published:** 2020-05-11

**Authors:** Fernando Azenha Bautzer Santos, Renan Barbosa Lemes, Paulo Alberto Otto

**Affiliations:** 1 Universidade de São Paulo, Instituto de Biociências, Departamento de Genética e Biologia Evolutiva, Laboratório de Genética Humana, São Paulo, SP, Brazil.

**Keywords:** Hardy-Weinberg proportions, computer program, Hardy Weinberg test

## Abstract

This article deals with a Windows (© Microsoft Inc.) executable, user-friendly program that tests the hypothesis of Hardy-Weinberg (HW) proportions from autosomal multiallelic data using different methods that include parametric, nonparametric and exact bootstrap tests, the latter obtained through computer simulations. The program can be obtained free of charge directly from the internet repository https://github.com/Lemes-RenanB/HardyWeinbergTesting.

In spite of its old age, the testing of Hardy-Weinberg proportions still is a kind of warhorse/cornerstone in population genetics. For example, the test is used extensively on a routine basis to exclude samples with gross molecular typing defects from the usually very large sets of genetic markers presently used in various types of population genetic analyses.

Still on the subject of growing old, the program presented here was the master thesis of the first author Santos (2006) and some of its features precede by at least two years the important contributions of Thomson *et al.* (2009) and Graffelman and Camarena (2008).

There exist many good population genetics programs, freely available in the internet; among other tasks, they provide adequate chi-squared and exact (computer simulated) testing for Hardy-Weinberg proportions. An in-depth analysis of the available computer programs for population genetics analysis is found in Excoffier and Heckel (2006), a review that certainly should be consulted in spite of being a bit outdated as to the program’s versions. Among the best and most used population genetic programs are the following: Arlequin (Excoffier *et al.*, 2005), PLINK (Purcell *et al.*, 2007), GENEPOP (Raymond and Rousset, 1995; Rousset, 2008), and PyPop (Lancaster *et al.*, 2003). These programs have a wide range of advanced, implemented methods related to population / molecular / medical genetics, so that that they require the insertion of specific instructions via command lines or data input in specific formats which can be a problem for users with minimal programming or computational skills. The testing of Hardy-Weinberg proportions is also provided by algorithms in different free, open access programming languages which demand from the users the command of their corresponding scripts and code editors. Examples are good population genetics packages written for Python or R, like the packages Genepop (a Python version of the the program GENEPOP) and the R packages genetics (Warnes and Warnes, 2007), adegenet (Jombart, 2008; Jombart and Ahmed, 2011), GAP (Zhao, 2007), and the Hardy-Weinberg R Package (Graffelman, 2015).

There exists also an incredible number of stand-alone computer programs that efficiently cope with the testing of HW proportions. Many among these programs can be executed directly from the www internet (as is the case of the excellent program HWDIAG, with its corresponding Bayesian assumptions detailed in Rogatko *et al.* (2002) while many others consist in executable software that can be freely downloaded into the user’s computers. Generally they test HW proportions using the usual chi-squared statistics, many of them performing (additionally or exclusively) simulations.

Given its specific scope (of dealing just with HW testing) and the simplicity of data input, our program is, in its turn, much simpler than the ones cited above, which makes it more user-friendly. In fact, its handling is so simple, direct and uncomplicated that it can be also used as a basic teaching tool in population genetics classes. Unlike other similar programs currently in use, our program tests the genotype proportions, in the two-allele case, using a large number of alternative methods; also in the two-allele case, the results are conveniently plotted using an alternate (isosceles) ternary system in which there is no distortion of genotype and allele frequencies. And, for the generalized case of any number of alleles segregating at an autosomal locus, besides the exact (bootstrap) probability of the null hypothesis of HW equilibrium, the program calculates exact (bootstrap) and approximate (Gaussian approximations for binomial variates) 95% confidence intervals for all the observed and expected frequencies of the possible genotypic classes. More importantly, people with only minimally basic computer skills can use our program without any difficulty, as (1) the data to be analyzed are directly entered in text boxes inside prompting windows in a graphic environment; and (2) the output images and texts can be easily obtained and stored.

The present program was adapted and updated from the original VISUAL BASIC (© Microsoft Inc.) version Santos (2006) and developed in Liberty BASIC v. 4.04, a dialect of BASIC language (© Shoptalk Systems 1992-2010, www.libertybasic.com) that runs in the PC Windows environment. The compressed, self-installing program can be obtained free of charge by email (lemes.rb@usp.br) or directly from the internet repository https://github.com/Lemes-RenanB/HardyWeinbergTesting. The program is the intellectual property of its authors, and as such, any use of it or of the materials included in it must contain an explicit reference to its origin. Feedback from users is welcome and will be used to improve the program and to correct unforeseen flaws. The program software is free and as such it comes with no warranty.

The program can be executed through the file HW_TEST.exe within the folder HW_TEST after unpacking the downloaded zipped file. The HW_TEST folder will contain the executable (compiled) file HW_TEST.exe, the corresponding application distribution (tokenized) file HW_TEST.tkn, a set of static and dynamic link library files necessary to run the compiled program (vbas31w.sll, vgui31w.sll, voflr31w.sll, vthk31w.dll, vtk1631w.dll, vtk3231w.dll, vvm31w.dll, and vvmt31w.dll). The user’s manual and the source code of the software (HW_TEST.bas) are also available in the same repository.

When the program icon (or the corresponding executable file) is activated, an interface graphic window will be displayed in the computer’s screen. If the number 2 is entered (two-allele case), the user is prompted with a two-allele window. The pdf user’s manual, besides reviewing in detail the statistical methods used, contains all figures showing in detail the program’s interface windows.

The text that follows corresponds to this two-allele case. The generalized case of n alleles will be dealt with at the end of this section.

The user should select, from the left-sided list on the two-allele window, the tests to be performed. The option ‘Chi-square without correction’ is pre-selected and will always be performed by the program. To run all available tests, the user should click the message bar “Select all”. The user should then enter on the right-sided genotype fields the absolute frequencies of observed genotypes to be tested. The program will not accept total sample sizes of observed data less than 5 or two null entries. There is no sense, either, in testing sample sizes of the order of 20 or less, because rarely the null hypothesis of HW ratios is rejected with such small sample sizes. Also, if the HW null hypothesis is rejected with sample sizes of this order of magnitude, the possibility of genotype misclassification typing errors should be seriously considered. Actually, the use of the present program in quality control of laboratory typing, even when dealing with larger sample sizes, is very important. Such deviations from HW proportions, besides resulting from low quality genotyping, could also be due to the effects of evolutionary selection processes, especially when limited to specific genomic regions (Weir, 2013).

The program performs the tests listed below (Yates, 1934; Hogben, 1946; Levene, 1949; Haldane, 1954; Cannings and Edwards, 1969; Weir, 1990). Summary explanations on the tests and other theoretical (mathematical/statistical) details are contained in the user’s pdf, which was adapted/updated/corrected from Santos (2006) and Otto (2008). Many of the topics discussed in the user’s pdf can also be found in the useful guidelines in Thomson *et al.* (2009).

1. Chi-squared HW tests with and without continuity correction
2. G (log-likelihood) tests with and without continuity correction
3. Fisher’s exact test
4. Hogben/Levene’s chi-squared method
5. Cannings & Edwards chi-squared method
6. Haldane’s exact test
7. exact test based on computer bootstrap simulations

The routine that performs the exact test based on computer bootstrap simulations starts by extracting the allele frequencies p and q of A and a alleles from the set of observed data (D = nAA, H = nAa, R = naa, D+H+R = N) and then calculates the probability of occurrence of the sample under the hypothesis of Hardy-Weinberg equilibrium:

P_0_ = N!/(2N)!.(nA!na!2^nAa^) /(nAA!nAa!naa!) = N!/(2N)!.(2D+H)!(H+2R)!2^H^ /(D!H!R!).

The program generates a normalized random number between 0 and 1; if the number is smaller than or equal to p^2^ = [(2D+H)/2N]^2^, this indicates that an AA homozygous genotype was obtained among the N of the sample; if the random number is larger than p^2^, but smaller than p^2^+2pq = 1-q^2^ = 1 - [(H+2R)/2N]^2^, this indicates that a heterozygous genotype Aa was generated; and, finally, if the random number is larger than 1-q^2^, the genotype is aa. The process is then repeated N-1 times, and in each instance the random number generated is compared to p^2^ and 1-q^2^. When the computer generates the N individuals of the sample, the frequencies p and q of A and a alleles are calculated from the numbers of AA-, Aa-, and aa-generated individuals. The computer repeats this process t times (t, the number of simulations is a number of the order of magnitude of 1,000 to 10,000; this program generates 1,000 simulations). After each simulation the computer calculates the value of the probability P_i_ of occurrence of the sample under the hypothesis of Hardy-Weinberg equilibrium:

P_i_ = N!/(2N)!.(2D_i_+H_i_)!(H_i_+2R_i_)!2^Hi^/(D_i_!H_i_!R_i_!).

This probability P_i_ is then compared to P_0_, the probability of occurrence of the observed sample under the hypothesis of HW equilibrium. The exact probability P, obtained after t simulations, is given by P = T/t (our program uses t = 1000 and P = T/1000), where T is the number of times in which P_i_ is smaller than or equal to P_0_.

A standard program text output obtained by running the program with the genotype data D = N(AA) = 119, H = N(Aa) = 42 and R = N(aa) = 39 and selecting all tests provided can be found in the user’s manual.

Besides generating 1,000 populations in expected HW proportions {P(AA) = p^2^, P(AB) = 2pq, P(BB) = q^2^}, the program simulates also 1,000 populations with frequencies {d = D/N, h = H/N, r = R/N}, where D, H and R are the observed numbers of sampled genotypes AA, AB and BB respectively. The two sets of scatter population points are then plotted (Figure 1) on an isosceles ternary diagram (Otto and Benedetti, 2000) that shows the HW parabola {p^2^, 2pq, q^2^} and its 95% chi-squared confidence intervals corresponding to the population of size N, represented by curves {p^2^ + pqF_LL_, 2pq(1-F_LL_), q^2^ + pqF_LL_} and {p^2^ + pqF_UL_, 2pq(1-F_UL_), q^2^ + pqF_UL_} with lower and upper limits F_LL_ = + √(3.841/N) and F_UL_ = - √(3.841/N) (Santos, 2006; Graffelman and Camarena, 2008).

**Figure 1:**
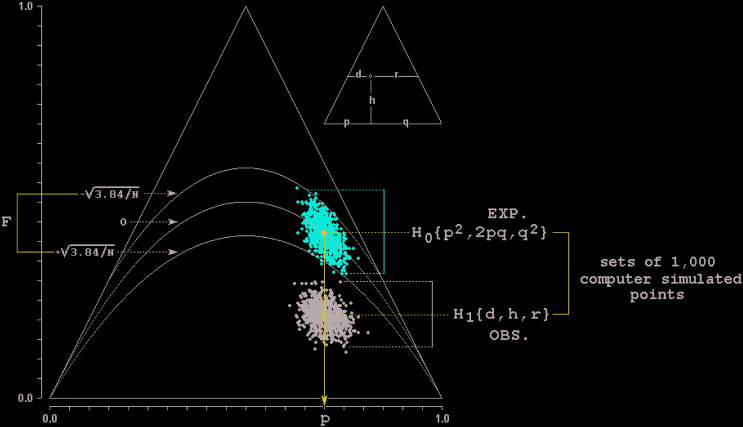
Trilinear diagram showing the results obtained with the observed sample N(AA) = 119, N(Aa) = 42, and N(aa) = 39. Please consult the article’s text (or the user’s manual) for explanations.

The program also calculates approximate 95% confidence intervals and exact confidence intervals of genotype frequencies, the latter based on algorithms that use random numbers to simulate genetic populations as described above.

If a number larger than 2 is entered (k-allele case) into the initial input window, the user is prompted with a k-allele window.

As in the two-allele case, in order to avoid running problems, the user should not use small total sample sizes (v.g. of the order of 10 k or less, where k is the number of alleles). Many null entries should also be avoided; in the common case when some of these occur, and especially when the total sample size is relatively small, the user is advised to properly agglutinate some value classes, thus reducing the number of alleles and improving the power of the test and the performance of the program. Just like in the two-allele case, there is no sense at all, either, in testing sample sizes of the order of 10 k or less, for the reasons already explained. And for any sample size, obtained test probability values of the order of less than 10^-6^ should be considered cautiously because they could result exclusively from genotyping errors with a large probability.

In the k-allele case the program performs the chi-squared test and the exact test based on computer bootstrap simulations.

As in the two-allele case, in the n-allele case Hardy-Weinberg (HW) equilibrium is usually a null hypothesis {H_0_: P(AA) = p_i_^2^, P(AB) = 2p_i_q_j_, ...} tested by Pearsons non-parametric chi-squared statistics χ^2^ = Σ(o_ij_ – e_ij_)^2^/e_ij_ = Σ(o_ij_^2^ / e_ij_) – N, where o_ij_ is the genotype observed absolute frequency, e_ij_ its corresponding expected figure in HW proportions, N the total sample size and the summation takes place from i = j = 1 to i = j = k. As there are k different alleles and the k(k+1)/2 expected genotype absolute frequencies are calculated conditional to the sample size N and to the fixed value of k-1 different allele frequencies extracted from the same sample, the number of degrees of freedom of the HW chi-squared test is calculated after k(k+1)/2 - k = k(k-1)/2. No continuity correction is applied to the test, since this is appropriate only for the two-allele case.

In the k-allele case, the program (a) performs a test based on computer bootstrap simulations, generating 1,000 populations in expected HW proportions, from which an exact P test probability is constructed; and (b) calculates approximate 95% confidence intervals and also exact confidence intervals of genotype frequencies, based on algorithms that use random numbers to simulate genetic populations (for details see the explanation in the corresponding paragraph of the item describing the simulation procedure in the 2-allele case).

As in the 2-allele case, an example standard text output (obtained by running the program with genotype data in the four-allele case and selecting the exact test option) is shown in the user’s manual.
